# Optimal Campaign in the Smoking Dynamics

**DOI:** 10.1155/2011/163834

**Published:** 2011-02-15

**Authors:** Gul Zaman

**Affiliations:** Centre for Advanced Mathematics and Physics, National University of Sciences and Technology, H-12, Islamabad 64000, Pakistan

## Abstract

We present the optimal campaigns in the smoking dynamics. Assuming that the giving up smoking model is described by the simplified PLSQ (potential-light-smoker-quit smoker) model, we consider two possible control variables in the form of education and treatment campaigns oriented to decrease the attitude towards smoking. In order to do this we minimize the number of light (occasional) and persistent smokers and maximize the number of quit smokers in a community. We first show the existence of an optimal control for the control problem and then derive the optimality system by using the Pontryagin maximum principle. Finally numerical results of real epidemic are presented to show the applicability and efficiency of this approach.

## 1. Introduction

 Application of control theory to epidemics is a very large field, and the study of epidemic models is strongly related to the possibility of evaluation of different control strategies: screening and educational campaigns [1], the vaccination campaign [2], and resource allocation [3]. A comprehensive survey of control theory applied to epidemiology was performed by Wickwire [4]. Many different models with different objective functions have been proposed (see [5–9]). A major difficulty in applying control theoretic methods to practical epidemiology problems is the commonly made assumption that one has total knowledge of the state of the epidemics [10].

In 2000, Castillo-Garsow et al. [11] for the first time proposed a simple mathematical model for giving up smoking. They consider a system with a total constant population which is divided into three classes: potential smokers, that is, people who do not smoke yet but might become smokers in the future (*P*), smokers (*S*), and people (former smokers) who have quit smoking permanently (*Q*). Sharomi and Gumel developed mathematical models by introducing mild and chain classes [12]. In their work they presented the development and public health impact of smoking-related illnesses. Zaman [13] extended the work of Castillo-Garsow et al. [11] and developed a model taking into account the occasional smokers compartment in the giving up smoking model and presented its qualitative behavior.

In this study we consider the model introduced by Zaman [13] and extend that once a smoker quits smoking he/she may become a potential smoker again and first discuss the dynamical behavior and then use optimal control theory for our optimal problem to minimize the number of light and persistent smokers and maximize the number of quit smokers in a community. In our control problem we first show the existence of an optimal control and then we derive the optimality system. The technical tool used in this work to determine the optimal strategy is the Pontryagin maximum principle. We emphasize that we have not set up a smoking model that particularly fit our optimization scheme. In order to do this we use optimal control strategies associated with two types of control, an education and a treatment campaigns to minimize the number of light and persistent smokers and maximize the number of quit smokers in a community. That is, we derive the optimality system consisting of the state and adjoint equations and solve numerically the system by using an iterative method. We also give an example of the real epidemic model by real data so that we illustrate how the optimal control theory can be applied in real situation.

The structure of this paper is organized as follows. First, we presented the qualitative behavior of the proposed model and then control system for the optimality and its existence, and the optimal control pairs are derived in Section 2. In Section 3, we solve numerically the optimality system by using real data, which consists of the original state system, the adjoint system, and their boundary conditions. Finally, we conclude by discussing results of the numerical simulation for our giving up smoking model.

## 2. Optimal Control Problem

In this section, we begin our explorations with a *PLSQ* model for a population as defined by the following system consisting of differential equations:

(1) $$\begin{array}{cc} \frac{dP\left(t\right)}{dt} & =bN\left(t\right)-\beta_{1}L\left(t\right)P\left(t\right)-\left(d_{1}+\mu \right)P\left(t\right)+\rho Q\left(t\right),\\ \frac{dL\left(t\right)}{dt} & =\beta_{1}L\left(t\right)P\left(t\right)-\beta_{2}L\left(t\right)S\left(t\right)-\left(d_{2}+\mu \right)L\left(t\right),\\ \frac{dS\left(t\right)}{dt} & =\beta_{2}L\left(t\right)S\left(t\right)-\left(\gamma +d_{3}+\mu \right)S\left(t\right),\\ \frac{dQ\left(t\right)}{dt} & =\gamma S\left(t\right)-\left(d_{4}+\mu +\rho \right)Q\left(t\right). \end{array}$$

As described in [13] the variables *P*, *L*, *S*, and *Q* represent the potential, light (occasional), persistent, and quit smoker, respectively. The parameter *b* represents the birth rate, *μ* is the natural death rate, and *γ* is the quit rate from smoking. The parameters *β*_1_ and *β*_2_ approximate the average number of contacts with smoker individuals needed to make light and persistent smoker, respectively, in each unit of time. *ρ* is the rate of quit smoker becoming potential smoker again, and *d*_1_ *,  d*_2_ *,  d*_3_, and *d*_4_ represent the death rate of potential smoker, occasional smoker, persistent smoker, and quit smoker, respectively. In this model, we also use the classic mass action hypothesis for both positive transmission coefficients *β*_1_ and *β*_2_. Potential smokers acquire infection at per capita rate *β*_1_ *L*(*t*). The total population size at time *t* is denoted by *N*(*t*) with *N*(*t*) = *P*(*t*) + *L*(*t*) + *S*(*t*) + *Q*(*t*). The dynamics of the total population are governed by the following differential equation:

(2) $$\begin{array}{c} \frac{dN}{dt}=\left(b-\mu \right)N\left(t\right)-\left(d_{1}P\left(t\right)+d_{2}L\left(t\right)+d_{3}S\left(t\right)+d_{4}Q\left(t\right)\right). \end{array}$$

If all individuals die at the same death rates, then we have *d*_1_ = *d*_2_ = *d*_3_ = *d*_4_ = *d*, and dynamics of the total population become

(3) $$\begin{array}{c} \frac{dN}{dt}=\left(b-d-\mu \right)N\left(t\right). \end{array}$$

When the birth rate is equal to the disease and natural death rate then the total population remains constant, that is, *b* = *d* + *μ*. The total constant population models were studied by several authors; see, for example, [8, 14]. In our proposed model it is not biologically feasible if we consider *b* < *d* + *μ*, so we assume that *b*⩾*d* + *μ*. The unique positive epidemic equilibrium of the system (1) is given by

(4) $$\begin{array}{cc} P^{⋆} & =\frac{\beta_{2}S^{⋆}+\left(d_{2}+\mu \right)}{\beta_{1}},\\ L^{⋆} & =\frac{\gamma +d_{3}+\mu }{\beta_{2}},\\ S^{⋆} & =\frac{\left(d_{2}+\mu \right)\left(d_{4}+\mu +\rho \right)\left(\beta_{1}\left(\gamma +d_{3}+\mu \right)+\beta_{2}\left(d_{1}+\mu \right)\right)}{\beta_{2}\left(\beta_{1}\rho \gamma -\beta_{1}\left(\gamma +d_{3}+\rho \right)-\beta_{2}\left(d_{1}+\mu \right)\left(d_{4}+\mu +\rho \right)\right)}\\ & \;\times \left(K-1\right),\\ Q^{⋆} & =\frac{\left(d_{2}+\mu \right)\left(\beta_{1}\left(\gamma +d_{3}+\mu \right)+\beta_{2}\left(d_{1}+\mu \right)\right)}{\beta_{2}\left(\beta_{1}\rho \gamma -\beta_{1}\left(\gamma +d_{3}+\rho \right)-\beta_{2}\left(d_{1}+\mu \right)\left(d_{4}+\mu +\rho \right)\right)}\\ & \;\times \left(K-1\right), \end{array}$$

where *K* = *bβ*_1_ *β*_2_ *N* ^⋆^/((*d*_2_ + *μ*)(*β*_1_(*γ* + *d*_3_ + *ρ*) + *β*_2_(*d*_1_ + *μ*))) represents the smoking generation number (basic reproductive number). It measures the average number of new smokers generated by single smoker in a population of potential smokers. The reproduction number is a concept in the epidemiology of infectious diseases [12]. It is a measure of how infectious a disease is and is required if you wish to calculate how many people you need to vaccinate if you are to achieve herd immunity. When somebody starts smoking, they may pass it on to nobody else or they may assist 1, 2, or more other people (who become secondary cases). The reproduction number, *K*, is the average (mean) number of secondary cases caused by each case of an infectious disease, during the infectious period. The number *K* will, of course, depend on a large number of factors depending on the states of the diseases. In our proposed model when *K* > 1 there exist persistent smokers in the community while for *K* < 1 there are no smokers.

In order to understand the qualitative behavior of the giving up smoking model we find the different equilibrium position of the model.

**Remark 1**

The Jacobian matrix around the trivial equilibrium *E*_*t*_ = (0,0, 0,0) is

(5) $$J_{t}=\left[\begin{array}{cccc} -d_{1}-\mu & 0 & 0 & \rho \\ 0 & -\mu -d_{2} & 0 & 0\\ 0 & 0 & -d_{3}-\gamma -\mu & 0\\ 0 & 0 & \gamma & -d_{4}-\mu -\rho \end{array}\right].$$

The eigenvalues of the Jacobian matrix around the trivial equilibrium *E*_0_ = (0,0, 0,0) are −*d*_1_ − *μ*, −*d*_2_ − *μ*, −*γ* − *d*_3_ − *μ*, −*d*_4_ − *μ* − *ρ*. Thus all the roots have negative real part, which shows that the trivial equilibrium is locally stable.

**Theorem 1**

When *K* > 1, the giving up smoking model (1) has unique positive epidemic equilibrium *E*_⋆_ = (*P* ^⋆^, *L* ^⋆^, *S* ^⋆^, *Q* ^⋆^), where *P* ^⋆^, *L* ^⋆^, *S* ^⋆^, and *Q* ^⋆^ are defined in (4).

**Theorem 2**

The giving up smoking model (1) has *E*_*l*_ = (1,0, 0,0) as a locally stable smoking-free equilibrium if and only if *β*_1_ < *d*_2_ + *μ*. Otherwise *E*_*l*_ is an unstable smoking-free equilibrium.

**Proof**

We can examine by linearizing our proposed giving up smoking model (1) around *E*_*l*_ = (1,0, 0,0) to get local stability. Hence, the equilibrium point around *E*_*l*_ gives us the Jacobian matrix:

(6) $$\begin{array}{c} J_{l}=\left[\begin{array}{cccc} -d_{1}-\mu & -\beta_{1} & 0 & \rho \\ 0 & \beta_{1}-\mu -d_{2} & 0 & 0\\ 0 & 0 & -d_{3}-\gamma -\mu & 0\\ 0 & 0 & \gamma & -d_{4}-\mu -\rho \end{array}\right]. \end{array}$$

The eigenvalues of the Jacobian matrix *J*_*l*_ around smoking-free equilibrium *E*_*l*_ = (1,0, 0,0) are −*d*_1_ − *μ*, *β*_1_ − *d*_2_ − *μ*, −*γ* − *d*_3_ − *μ*, −*d*_4_ − *μ* − *ρ*. Thus, we deduce that all the roots have negative real part when *β*_1_ < *d*_2_ + *μ* which shows that the smoking-free equilibrium is locally asymptotically stable.

**Theorem 3**

When *K* > 1, the unique positive epidemic equilibrium *E*_⋆_ = (*P* ^⋆^, *L* ^⋆^, *S* ^⋆^, *Q* ^⋆^) of the giving up smoking model (1) is locally asymptotically stable.

**Proof**

For the proof of this theorem see Appendix A

In order to investigate an effective campaign to control smoking in a community which satisfies that the maximum number of occasional (light) and persistent smoker individuals is not larger than that of potential smoker individuals and more individuals are recovered after quitting the use of tobacco, we consider the system (1) and use two control variables to control both the occasional (light) smoker and persistent smoker population. In the system (1) we have four state variables *P*(*t*), *L*(*t*), *S*(*t*), and *Q*(*t*). For the optimal control problem we consider the control variable *u*(*t*) = (*u*_1_(*t*), *u*_2_(*t*)) ∈ *U* relative to the state variables (*P*(*t*), *L*(*t*), *S*(*t*), *Q*(*t*)), where *U* = {(*u*_1_, *u*_2_) | *u*_*i*_(*t*) is measurable and 0 ≤ *u*_*i*_(*t*) ≤ *ω*_*i*_ < *∞*, *t* ∈ [0, *t*_end_], for *i* = 1,2, }, is an admissible control set.

Next, we describe the role played by the first control *u*_1_(*t*) and how it is incorporated in the system (1). It the beginning people start smoking occasionally for a variety of different reasons. Some think it looks cool. Others start because their family members or friends smoke. Statistics show that about 9 out of 10 tobacco users start occasionally and then gradually become persistent smoker. Furthermore there are strong evidence that the attitude towards smoking is starting from high (junior) school time. Thus, a campaign like movie particularly in a class or group to show them that smoking is more likely to develop cancer of the mouth and throat, bladder, kidney, liver, stomach, and pancreas which significantly raises risk of heart attack. Therefore control function *u*_1_(*t*) represents education campaign level used to control smoking in a community. The control *u*_2_(*t*) represents the level of treatment in the form of stop smoking injection (vaccine) that contains drugs that block nicotine receptors of the brain, which in turn helps in reducing the desire to smoke. The physical meaning of the control variable in this problem is that low levels of the number of occasional (light) and persistent smokers build. In case of no campaign or no treatment the number of persistent smoker increases while the number of former smoker decreases. Better and prefect time of campaign brings the number of occasional (light) and persistent smokers to a small level, potential smokers begin to build again and more individuals are recovered by quitting the use of tobacco. The effects of tobacco on both occasional (light) and persistent smokers are negative for quit smokers around them, so we wish to minimize both. Also small amount of control variables (campaign and treatment) are acceptable; therefore, we wish to penalize for amount too large, so quadratic terms for both control variables will be analyzed. Hence, our optimal control problem to minimize the objective functional is given by

(7) $$J\left(u_{1},u_{2}\right)=\overset{T}{\underset{0}{\int }}\left[\alpha_{1}L\left(t\right)+\alpha_{2}S\left(t\right)+\frac{1}{2}\left(\xi_{1}u_{1}^{2}\left(t\right)+\xi_{2}u_{2}^{2}\left(t\right)\right)\right]dt$$

subject to

(8) $$\begin{array}{cc} \frac{dP\left(t\right)}{dt} & =bN\left(t\right)-\beta_{1}L\left(t\right)P\left(t\right)-\left(d_{1}+\mu \right)P\left(t\right)+\rho Q\left(t\right),\\ \frac{dL\left(t\right)}{dt} & =\beta_{1}L\left(t\right)P\left(t\right)-\beta_{2}L\left(t\right)S\left(t\right)-\left(d_{2}+\mu +u_{1}\left(t\right)\right)L\left(t\right),\\ \frac{dS\left(t\right)}{dt} & =\beta_{2}L\left(t\right)S\left(t\right)-\left(\gamma +d_{3}+\mu +u_{2}\left(t\right)\right)S\left(t\right),\\ \frac{dQ\left(t\right)}{dt} & =\left(\gamma +u_{2}\left(t\right)\right)S\left(t\right)-\left(d_{4}+\mu +\rho \right)Q\left(t\right)+u_{1}\left(t\right)L\left(t\right), \end{array}$$

with initial conditions

(9) $$\begin{array}{c} P\left(0\right)=P_{0},\;\;L\left(0\right)=L_{0},\;\;S\left(0\right)=S_{0},\;\;Q\left(0\right)=Q_{0}. \end{array}$$

Here *α*_*i*_ and *ξ*_*i*_ for *i* = 1,2 are weight factors (positive constants) that represent a smoker level of acceptance of the control campaign. The goal is to minimize the occasional (light) and persistent smokers and the cost of implementing the control. Occasional smokers induce an optimal control *u*_1_(*t*) before they become persistent smokers, and an optimal control *u*_2_(*t*) should be provided to persistent smokers population.

Various deterministic optimal control models and their existence are investigated by several authors [8, 9, 15]. First we shall show the existence for the control system (8). Let *P*(*t*), *L*(*t*), *S*(*t*), and *Q*(*t*) be state variables with control variable *u*(*t*) = (*u*_1_(*t*), *u*_2_(*t*)) ∈ *U*. For existence we consider the control system (8) with initial conditions in (9). Then we can rewrite the system (8) in the following form:

(10) $$\begin{array}{c} V_{t}=MV+\chi \left(V\right), \end{array}$$

where

(11) $$\begin{array}{c} V=\left[\begin{array}{c} P\left(t\right)\\ L\left(t\right)\\ S\left(t\right)\\ Q\left(t\right) \end{array}\right],\;\;M=\left[\begin{array}{cccc} -d_{1}-\mu & 0 & 0 & \rho \\ 0 & -\left(d_{2}+\mu +u_{1}\left(t\right)\right) & 0 & 0\\ 0 & 0 & -\left(\gamma +d_{3}+\mu +u_{2}\left(t\right)\right) & 0\\ 0 & u_{1}\left(t\right) & \gamma +u_{2}\left(t\right) & -\left(d_{4}+\mu +\rho \right) \end{array}\right],\\ \chi \left(V\right)=\left[\begin{array}{c} bN\left(t\right)-\beta_{1}P\left(t\right)L\left(t\right)\\ \beta_{1}P\left(t\right)L\left(t\right)+\beta_{2}L\left(t\right)S\left(t\right)\\ \beta_{2}L\left(t\right)S\left(t\right)\\ 0 \end{array}\right], \end{array}$$

and *V*_*t*_ denotes the derivative of *V* with respect to the time *t*. The system (10) is a nonlinear system with a bounded coefficient. We set

(12) $$\begin{array}{c} G\left(V\right)=MV+\chi \left(V\right). \end{array}$$

The second term on the right-hand side of (12) satisfies

(13) $$\begin{array}{cc} \left|\chi \left(V_{1}\right)-\chi \left(V_{2}\right)\right| & \leq \Lambda_{1}\left|P_{1}\left(t\right)-P_{2}\left(t\right)\right|+\Lambda_{2}\left|L_{1}\left(t\right)-L_{2}\left(t\right)\right|\\ & \;+\Lambda_{3}\left|S_{1}\left(t\right)-S_{2}\left(t\right)\right|, \end{array}$$

where the positive constants Λ_1_, Λ_2_, and Λ_3_ are independent of state variables *P*(*t*), *L*(*t*), and *S*(*t*) ≤ *N*(*t*), respectively. Also we get

(14) $$\begin{array}{c} \left|G\left(V_{1}\right)-G\left(V_{2}\right)\right|\leq \Lambda \left|V_{1}-V_{2}\right|, \end{array}$$

where Λ = Λ_1_ + Λ_2_ + Λ_3_ + ||*M*||<*∞*. Thus it follows that the function *G* is uniformly Lipschitz continuous. From the definition of *U* and the restriction on *P*(*t*), *L*(*t*), *S*(*t*), and *Q*(*t*) ≥ 0 we can see that a solution of the system (10) exists (see [16]).

To illustrate how to solve a control problem actually, first we should find Hamiltonian for the optimal control problem (7)–(9). To do this, we define the Hamiltonian *H* for the control problem as follows:

(15) $$\begin{array}{cc} & H\left(P,L,S,Q,u_{1},u_{2},\lambda_{1},\lambda_{2},\lambda_{3},\lambda_{4}\right)\\ & \;\;=Ł\left(L,S,u_{1},u_{2}\right)+\lambda_{1}\left(t\right)g_{1}\left(t\right)+\lambda_{2}\left(t\right)g_{2}\left(t\right)+\lambda_{3}\left(t\right)g_{3}\left(t\right), \end{array}$$

where *Ł*(*L*, *S*, *u*_1_, *u*_2_) = *α*_1_ *L*(*t*) + *α*_2_ *S*(*t*) + (1/2)(*ξ*_1_ *u*_1_^2^(*t*) + *ξ*_2_ *u*_2_^2^(*t*)) is the Lagrangian and *g*_*i*_ for *i* = 1,2, 3 is the right-hand side of the differential equations of the state system (8), respectively.

**Theorem 4**

There exists an optimal control pair u* = (*u*_1_*, *u*_2_*) ∈ *U* such that

(16) $$\begin{array}{c} J\left(u_{1}^{*},u_{2}^{*}\right)=\underset{\left(u_{1},u_{2}\right)\in U}{\min\;}J\left(u_{1},u_{2}\right), \end{array}$$

subject to the control system (8) with initial conditions (9).

**Proof**

To prove the existence of an optimal control, we have to show the following.

(1) The control and state variables are nonnegative values.
(2) The control *U* set is convex and closed.
(3) The RHS of the state system is bounded by linear function in the state and control variables.
(4) The integrand of the objective functional is concave on *U*.
(5) There exist constants such that the integrand in (7) of the objective functional is satisfied.

In order to verify these conditions, we use a result by Fister [5]. We note that the solutions are bounded. The set of all the control variables *u*(*t*) = (*u*_1_(*t*), *u*_2_(*t*)) ∈ *U*, is also convex and closed by definition. The optimal system is bounded which determines the compactness needed for the existence of the optimal control. In addition, the integrand in functional (7) is given by *α*_1_ *L*(*t*) + *α*_2_ *S*(*t*) + (1/2)(*ξ*_1_ *u*_1_^2^(*t*) + *ξ*_2_ *u*_2_^2^(*t*)) and is convex on the control set *U*. Also we can easily see that there exist a constant *σ* > 1 and positive numbers *η*_1_ and *η*_2_ such that

(17) $$\begin{array}{c} J\left(u_{1},u_{2}\right)\geq \eta_{2}+\eta_{1}{\left({\left|u_{1}\right|}^{2}+{\left|u_{2}\right|}^{2}\right)}^{\sigma /2}, \end{array}$$

which completes the existence of an optimal control.

Pontryagin maximum principal (PMP) states necessary conditions [17] that must hold on an optimal trajectory. These conditions help us in calculation of the state, adjoint, and control variables in the optimal control problems. PMP can be used as both a computational technique and analytic technique. The technical tools used to determine the optimal strategy is given in the following form.

If (x*(*t*), u*(*t*)) is an optimal solution of an optimal control problem, then there exists a nontrivial vector function *λ*(*t*) = (*λ*_1_(*t*), *λ*_2_(*t*),…, *λ*_*n*_(*t*)) satisfying the following inequalities:

(18) $$\begin{array}{cc} x^{\prime }\left(t\right) & =\frac{\partial H\left(t,x^{*}\left(t\right),u^{*}\left(t\right),\lambda \left(t\right)\right)}{\partial \lambda },\\ 0 & =\frac{\partial \text{H}\left(t,x^{*}\left(t\right),u^{*}\left(t\right),\lambda \left(t\right)\right)}{\partial u},\\ \lambda^{\prime }\left(t\right) & =-\frac{\partial H\left(t,x^{*}\left(t\right),u^{*}\left(t\right),\lambda \left(t\right)\right)}{\partial x}, \end{array}$$

where “′” denotes the derivative with respect to time *t*. Now we apply the necessary conditions to the Hamiltonian *H* in (15).

**Theorem 5**

Let P*(*t*), L*(*t*), S*(*t*), and Q*(*t*) be optimal state solutions with associated optimal control variables *u*_1_*(*t*) and *u*_2_*(*t*) for the optimal control problem (7)–(9). Then there exist adjoint variables *λ*_1_(*t*), *λ*_2_(*t*), *λ*_3_(*t*), and *λ*_4_(*t*) satisfing

(19) $$\begin{array}{cc} {\lambda^{\prime }}_{1}\left(t\right) & =\beta_{1}\left(\lambda_{1}\left(t\right)-\lambda_{2}\left(t\right)\right)L^{*}\left(t\right)+\lambda_{1}\left(t\right)\left(d_{1}+\mu \right),\\ {\lambda^{\prime }}_{2}\left(t\right) & =\beta_{1}\left(\lambda_{1}\left(t\right)-\lambda_{2}\left(t\right)\right)P^{*}\left(t\right)+\beta_{2}\left(\lambda_{2}\left(t\right)-\lambda_{3}\left(t\right)\right)S^{*}\left(t\right)\\ & \;+\lambda_{2}\left(t\right)\left(d_{2}+\mu \right)+\left(\lambda_{2}\left(t\right)-\lambda_{4}\left(t\right)\right)u_{1}\left(t\right)-\alpha_{1},\\ {\lambda^{\prime }}_{3}\left(t\right) & =\beta_{2}\left(\lambda_{2}\left(t\right)-\lambda_{3}\left(t\right)\right)L^{*}\left(t\right)+\lambda_{3}\left(t\right)\left(\gamma +d_{3}+\mu \right)\\ & \;+\left(\lambda_{3}\left(t\right)-\lambda_{4}\left(t\right)\right)u_{2}\left(t\right)-\alpha_{2},\\ {\lambda \prime }_{4}\left(t\right) & =\left(\lambda_{4}\left(t\right)-\lambda_{1}\left(t\right)\right)\rho +\lambda_{4}\left(t\right)\left(d_{4}+\mu \right), \end{array}$$

with transversality conditions (or boundary conditions)

(20) $$\begin{array}{c} \lambda_{i}\left(T\right)=0,\;i=1,2,3. \end{array}$$

Furthermore, optimal control pairs are given as follows:

(21) $$\begin{array}{c} u_{1}^{*}\left(t\right)=\max\;\left\{\min\;\left\{\frac{\left(\lambda_{1}\left(t\right)-\lambda_{3}\left(t\right)\right)L\left(t\right)}{\xi_{1}},\omega_{1}\right\},0\right\}, \end{array}$$

(22) $$\begin{array}{c} u_{2}^{*}\left(t\right)=\max\;\left\{\min\;\left\{\frac{\left(\lambda_{2}\left(t\right)-\lambda_{3}\left(t\right)\right)S\left(t\right)}{\xi_{2}},\omega_{2}\right\},0\right\}. \end{array}$$

**Proof**

For the proof of this theorem see Appendix B.

Here we call formulas (21) and (22) for u* the characterization of the optimal control. The optimal control and the state are found by solving the optimality system, which consists of the state system (8), the adjoint system (20), boundary conditions (9) and (20), and the characterization of the optimal control (21) and (22). To solve the optimality system we use the initial and transversality conditions together with the characterization of the optimal control pair (*u*_1_*(*t*), *u*_2_*(*t*)) given by (21) and (22). By substituting the values of *u*_1_*(*t*) and *u*_2_*(*t*) in the control system (8) we get the following system:

(23) $$\begin{array}{cc} & \frac{dP^{*}\left(t\right)}{dt}\\ & =bN^{*}\left(t\right)-\beta_{1}L^{*}\left(t\right)P^{*}\left(t\right)-\left(d_{1}+\mu \right)P^{*}\left(t\right)+\rho Q^{*}\left(t\right),\\ & \frac{dL^{*}\left(t\right)}{dt}\\ & =\beta_{1}L^{*}\left(t\right)P^{*}\left(t\right)-\beta_{2}L^{*}\left(t\right)S^{*}\left(t\right)\\ & \;-\left(d_{2}+\mu +\max\;\left\{\min\;\left\{\frac{\left(\lambda_{2}\left(t\right)-\lambda_{4}\left(t\right)\right)L^{*}\left(t\right)}{\xi_{1}},\omega_{1}\right\},0\right\}\right)\\ & \;\times L^{*}\left(t\right),\\ & \frac{dS^{*}\left(t\right)}{dt}\\ & =\xi_{2}L^{*}\left(t\right)S^{*}\left(t\right)\\ & \;-\left(\gamma +d_{3}+\mu +\max\;\left\{\min\;\left\{\frac{\left(\lambda_{3}\left(t\right)-\lambda_{4}\left(t\right)\right)S^{*}\left(t\right)}{\xi_{2}},\omega_{2}\right\},0\right\}\right)\\ & \;\times S^{*}\left(t\right),\\ & \frac{dQ^{*}\left(t\right)}{dt}\\ & =\left(\gamma +\max\;\left\{\min\;\left\{\frac{\left(\lambda_{3}\left(t\right)-\lambda_{4}\left(t\right)\right)S^{*}\left(t\right)}{\xi_{2}},\omega_{2}\right\},0\right\}\right)S^{*}\left(t\right)\\ & \;-\left(d_{4}+\mu +\rho \right)Q^{*}\left(t\right)\\ & \;+\max\;\left\{\min\;\left\{\frac{\left(\lambda_{2}\left(t\right)-\lambda_{4}\left(t\right)\right)L^{*}\left(t\right)}{\xi_{1}},\omega_{1}\right\},0\right\}L^{*}\left(t\right). \end{array}$$

With the Hamiltonian H* at (P*,L*, S*, Q*, *u*_1_*, *u*_2_*, *λ*_1_, *λ*_2_, *λ*_3_, *λ*_4_),

(24) $$\begin{array}{cc} & H^{*}\\ & =\alpha_{1}L^{*}\left(t\right)+\alpha_{2}S^{*}\left(t\right)\\ & \;+\frac{1}{2}[\xi_{1}{\left(\max\;\left\{\min\;\left\{\frac{\left(\lambda_{2}\left(t\right)-\lambda_{4}\left(t\right)\right)L^{*}\left(t\right)}{\xi_{1}},\omega_{1}\right\},0\right\}\right)}^{2}\\ & \;\;\;\;\;+\xi_{2}{\left(\max\;\left\{\min\;\left\{\frac{\left(\lambda_{3}\left(t\right)-\lambda_{4}\left(t\right)\right)S^{*}\left(t\right)}{\xi_{2}},\omega_{2}\right\},0\right\}\right)}^{2}]\\ & \;+\lambda_{1}\left(t\right)g_{1}^{*}\left(t\right)+\lambda_{2}\left(t\right)g_{2}^{*}\left(t\right)+\lambda_{3}\left(t\right)g_{3}^{*}\left(t\right), \end{array}$$

where *g*_i_*(*t*) for *i* = 1,2, 3 is the right-hand side of the differential equations of the state system (23), respectively. To find out the optimal control and state we will numerically solve the system (23) and Hamiltonian (24).

## 3. Numerical Results

In this section, we shall solve numerically an optimal control problem for the *PLSQ* model. Here we obtain the optimality system from the state and adjoint equations. The optimal control problem strategy is obtained by solving the optimal system, which consists of eight ordinary differential equations and boundary conditions. The optimality system can be solved by using an iterative method by Runge-Kutta fourth scheme [18]. Using an initial guess for the control variables, *u*_1_(*t*) and *u*_2_(*t*), the state variables, *P*, *L*, *S*, and *Q* are solved forward in time, and then the adjoint variables, *λ*_*i*_ for *i* = 1,2, 3,4, are solved backwards in time. If the new values of the state and adjoint variables differ from the previous values, the new values are used to update *u*_1_(*t*) and *u*_2_(*t*), and the process is repeated until the system converges. For the *PLSQ* model presented in this work, the state system is given by (8) with the initial conditions given by (9). The adjoint system is given by (20) with the final time conditions given by (21) and the characterization of the optimal control by (21) and (22).

To determine the numerical simulation of the optimality system we use a small time step size Δ*t* = 0.005. We consider that the optimal campaign continues for 30 day, and use a real estimate of parameter value represented in Table 1. For reasonable values of the parameter we restate the idea presented in [8, 13, 19]. When a person first becomes a smoker it is not likely that she/he quits for several years since tobacco contains nicotine, which is shown to be an addictive drug. We assume 1/*γ* to be a value between 15 and 25 years, that is, 20. Hence, the value of 1/*γ* is set to 7300 days. The death rate of each individual is different from others and depends on the real life situation. Here, we assume that *d*_4_ ≤ *d*_1_ ≤ *d*_2_ ≤ *d*_3_. The natural death rate *μ* is per 1000 per year (currently 8 in the US). There is strong evidence that the attitude towards smoking is starting from high (junior) school time. Therefore 1/*b* is set to be 1095 days. Death from lung cancer was the leading cause by smoking, with a rate of 37 per 100,000 individuals [20]. The reasonable value of parameters *β*_1_ and *β*_2_ which represent the average number of contacts with smoker individuals needed to make light and persistent smoker, respectively, in each unit of time can be obtained by using the same techniques presented in [13]. We consider the real data used in [8] for the *P*(0) = 153,  *S*(0) = 79, and assume that *L*(0) = 40. and *Q*(0) = 9.

**Figure 1:**
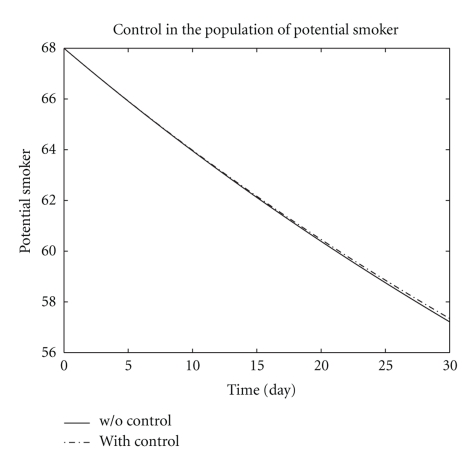
The plot shows the population of potential smokers *P* both with control and without control.

**Figure 2:**
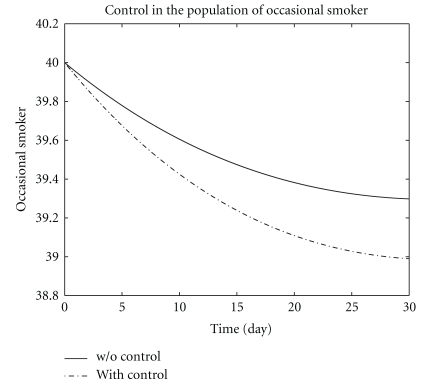
The plot shows occasional smoker *L* both with control and without control.

We presented in Figure 1 the potential smoker in two systems (1) and (8). The plain line denotes the population of potential smokers in the system (1) without control while the dotted line denotes the population of potential smokers in the system (8) with control. The potential smokers in the two systems (1) and (8) are almost the same in around days 1–10. After 10 days a slight increase appears in potential smokers population.  Figure 2 represents the population of occasional smokers in two systems (1) without control and (8) with control. The population of occasional smokers sharply decreases from the first day of infection in systems both with control and without control and reaches its minimum number of occasional smokers *L* = 39.3 and L* = 39 at the end of the optimal campaign. 

**Figure 3:**
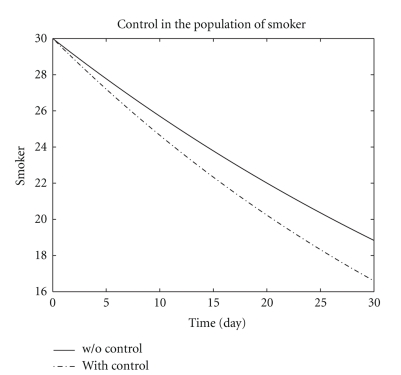
The plot shows persistent smoker *S* both with control and without control.

**Figure 4:**
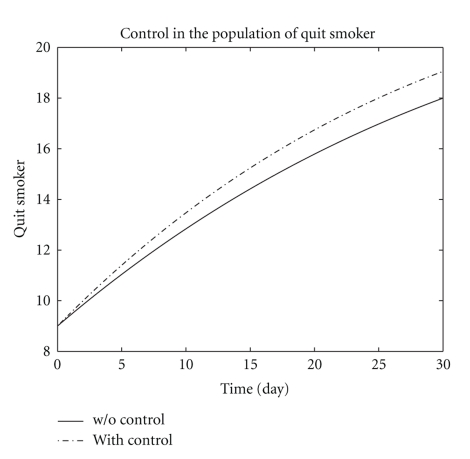
The plot shows quit smoker *Q* both with control and without control.

Figure 3 represents the persistent smokers in the two systems (1) and (8). The persistent smokers population in both systems (1) and (8) decreases more sharply than the light (occasional) smokers population and about *S* = 19 and S* = 16.8 persistent smokers population remains at the end of the optimal campaign.  Figure 4 represents the numerical results of both systems (1) and (8) of the quit smokers population. After the optimal campaign the light (occasional) smokers and persistent smokers populations decreases while the quit smoker population increases.

In this work we use one set of parameters for both dynamical systems (1) and (8). Simulations with different sets of parameter values can be used in the future to obtain a sampling of possible behaviors of a dynamical system.

## 4. Conclusion

 In this paper we considered the model introduced by Zaman [13] and extend that once a smoker quits he/she may becomes a potential smoker again, and then we used optimal control theory to minimize the number of light and persistent smokers and maximize the number of quit smokers in a community. The control plots obtained from the numerical simulation in this paper represent that the numbers of light and persistent smokers decreases and the number of quit smokers increases in the optimality system. We also showed that for certain values of control rate there exists its corresponding optimal solution. We considered a special disease (smoking) in a specific community as a realistic model, and we hope that the approach introduced in this paper will be applicable in other epidemic models beyond the giving up smoking model.

## Figures and Tables

**Table 1:** Reasonable values of the parameter.

Parameter	Description	Value
*b*	Birth rate	0.00091
*μ*	Natural death rate	0.0031
*γ*	Recovery rate	0.0013
*ρ*	The rate of *Q* becomes *P* again	0.0031
*β*_1_	Infection rate of smoking	0.00014
*β*_2_	Infection rate of smoking	0.0024
*α*_1_	Weight factor for smoker	0.091
*α*_2_	Weight factor for smoker	0.001
*ξ*_1_	Campaign level of acceptance for smoker	0.02
*ξ*_2_	Treatment level of acceptance for smoker	0.10
*d*_1_	Disease death rate of *P *	0.00034
*d*_2_	Disease death rate of *L *	0.00045
*d*_3_	Disease death rate of *S *	0.0054
*d*_4_	Disease death rate of *Q *	0.00061

## Appendices

### A. Proof of Theorem 3

The Jacobian matrix of the giving up smoking model (1) around *E*_⋆_ = (*P* ^⋆^, *L* ^⋆^, *S* ^⋆^, *Q* ^⋆^) is

(A.1) $$\begin{array}{c} J^{⋆}=\left[\begin{array}{cccc} A_{P} & A_{L} & 0 & \rho \\ B_{P} & B_{L} & B_{S} & 0\\ o & C_{L} & C_{S} & 0\\ 0 & 0 & \gamma & D_{Q} \end{array}\right], \end{array}$$

where

(A.2) $$\begin{array}{c} A_{P}=-\beta_{1}L^{⋆}-\left(d_{1}+\mu \right),\;\;A_{L}=-\beta_{1}P^{⋆},\\ B_{P}=\beta_{1}L^{⋆},\;\;B_{P}=\beta_{1}P^{⋆}-\beta_{2}S^{⋆}-\left(d_{2}+\mu \right),\\ B_{S}=-\beta_{2}L^{⋆},\;\;C_{L}=\beta_{2}S^{⋆},\\ C_{S}=\beta_{2}L^{⋆}-\left(\gamma +d_{3}+\mu \right),\;\;D_{Q}=-\left(d_{4}+\mu +\rho \right). \end{array}$$

The characteristic equation of *J* ^⋆^ is

(A.3) $$\begin{array}{c} \lambda^{4}+a_{1}\lambda^{3}+a_{2}\lambda^{2}+a_{3}\lambda +a_{4}=0, \end{array}$$

where

(A.4) $$\begin{array}{cc} a_{1} & =-\left(A_{P}+B_{L}+C_{S}+D_{Q}\right),\\ a_{2} & =C_{S}D_{Q}+A_{P}B_{C}+\left(C_{S}+D_{Q}\right)\left(A_{P}+B_{L}\right)-A_{L}B_{P},\\ a_{3} & =A_{L}B_{P}\left(C_{S}+D_{Q}\right)-\left(C_{S}D_{Q}\left(A_{P}+B_{L}\right)+A_{P}B_{C}\left(C_{S}+D_{Q}\right)\right),\\ a_{4} & =A_{P}B_{L}C_{S}D_{Q}-A_{L}B_{P}C_{S}D_{Q}-\rho \gamma B_{P}C_{L}. \end{array}$$

By Routh-Hurwitz conditions the equilibrium state is asymptotically stable if *a*_1_ > 0, *a*_3_ > 0, *a*_4_ > 0 and *a*_1_ *a*_2_ *a*_3_ > *a*_3_^2^ + *a*_1_^2^ *a*_4_. Thus it follows that the unique positive equilibrium *E*_⋆_ of the giving up smoking model, which exists if *K* > 1, is always locally asymptotically stable. This completes the proof.

### B. Proof of Theorem 5

To determine the adjoint equations and the transversality conditions, we use Hamiltonian (15). From setting *P*(*t*) = *P**(*t*), *L*(*t*) = *L**(*t*), *S*(*t*) = *S**(*t*), and *Q*(*t*) = *Q**(*t*) and differentiating the Hamiltonian (15) with respect to *P*, *L*, *S*, and *Q*, we obtain

(B.1) $$\begin{array}{cc} \lambda_{1}^{\prime }\left(t\right) & =-\frac{\partial H}{\partial P}=\beta_{1}\left(\lambda_{1}\left(t\right)-\lambda_{2}\left(t\right)\right)L\left(t\right)+\lambda_{1}\left(t\right)\left(d_{1}+\mu \right),\\ \lambda_{2}^{\prime }\left(t\right) & =-\frac{\partial H}{\partial L}\\ & =\beta_{1}\left(\lambda_{1}\left(t\right)-\lambda_{2}\left(t\right)\right)P\left(t\right)\\ & \;+\beta_{2}\left(\lambda_{2}\left(t\right)-\lambda_{3}\left(t\right)\right)S\left(t\right)+\lambda_{2}\left(t\right)\left(d_{2}+\mu \right)\\ & \;+\left(\lambda_{2}\left(t\right)-\lambda_{4}\left(t\right)\right)u_{1}\left(t\right)-\alpha_{1},\\ \lambda_{3}^{\prime }\left(t\right) & =-\frac{\partial H}{\partial S}\\ & =\beta_{2}\left(\lambda_{2}\left(t\right)-\lambda_{3}\left(t\right)\right)L\left(t\right)+\lambda_{3}\left(t\right)\left(\gamma +d_{3}+\mu \right)\\ & \;+\left(\lambda_{3}\left(t\right)-\lambda_{4}\left(t\right)\right)u_{2}\left(t\right)-\alpha_{2},\\ \lambda_{4}^{\prime }\left(t\right) & =-\frac{\partial H}{\partial Q}=\left(\lambda_{4}\left(t\right)-\lambda_{1}\left(t\right)\right)\rho +\lambda_{4}\left(t\right)\left(d_{4}+\mu \right). \end{array}$$

Using the optimality conditions and the property of the control space *U* for the control variable *u*_1_, we obtain

(B.2) $$\begin{array}{c} \frac{\partial H}{\partial u_{1}}=0\Rightarrow \xi_{1}u_{1}\left(t\right)+\left(-\lambda_{2}\left(t\right)+\lambda_{4}\left(t\right)\right)L\left(t\right)=0. \end{array}$$

By using the property of the control space, we obtain

(B.3) $$\begin{array}{c} u_{1}^{*}\left(t\right)=\{\begin{array}{cc} 0 & \text{if}\;\frac{\left(\lambda_{2}\left(t\right)-\lambda_{4}\left(t\right)\right)L\left(t\right)}{\xi_{1}}\leq 0,\;\\ \frac{\left(\lambda_{2}\left(t\right)-\lambda_{4}\left(t\right)\right)L\left(t\right)}{\xi_{1}} & \text{if}\;0<\frac{\left(\lambda_{2}\left(t\right)-\lambda_{4}\left(t\right)\right)L\left(t\right)}{\xi_{1}},<\omega_{1}\\ \omega_{1} & \text{if}\;\frac{\left(\lambda_{2}\left(t\right)-\lambda_{4}\left(t\right)\right)L\left(t\right)}{\xi_{1}}\geq \omega_{1}. \end{array} \end{array}$$

This can be rewritten in compact notation

(B.4) $$\begin{array}{c} u_{1}^{*}\left(t\right)=\max\;\left\{\min\;\left\{\frac{\left(\lambda_{2}\left(t\right)-\lambda_{4}\left(t\right)\right)L\left(t\right)}{\xi_{1}},\omega_{1}\right\},0\right\}. \end{array}$$

Similar to the control variable *u*_2_, we get

(B.5) $$\begin{array}{c} u_{2}^{*}\left(t\right)=\{\begin{array}{cc} 0 & \text{if}\;\frac{\left(\lambda_{3}\left(t\right)-\lambda_{4}\left(t\right)\right)S\left(t\right)}{\xi_{2}}\leq 0,\\ \frac{\left(\lambda_{3}\left(t\right)-\lambda_{4}\left(t\right)\right)S\left(t\right)}{\xi_{2}} & \text{if}\;0<\frac{\left(\lambda_{3}\left(t\right)-\lambda_{4}\left(t\right)\right)S\left(t\right)}{\xi_{2}}<\omega_{2},\\ \omega_{2} & \text{if}\;\frac{\left(\lambda_{3}\left(t\right)-\lambda_{4}\left(t\right)\right)S\left(t\right)}{\xi_{2}}\geq \omega_{2}. \end{array} \end{array}$$

This can also be rewritten similar to the above compact form as

(B.6) $$\begin{array}{c} u_{2}^{*}\left(t\right)=\max\;\left\{\min\;\left\{\frac{\left(\lambda_{3}\left(t\right)-\lambda_{4}\left(t\right)\right)S\left(t\right)}{\xi_{2}},\omega_{2}\right\},0\right\}. \end{array}$$

This completes the proof.
